# Identification of Gradient Promoters of *Gluconobacter oxydans* and Their Applications in the Biosynthesis of 2-Keto-L-Gulonic Acid

**DOI:** 10.3389/fbioe.2021.673844

**Published:** 2021-04-09

**Authors:** Yue Chen, Li Liu, Shiqin Yu, Jianghua Li, Jingwen Zhou, Jian Chen

**Affiliations:** ^1^Key Laboratory of Industrial Biotechnology, Ministry of Education and School of Biotechnology, Jiangnan University, Wuxi, China; ^2^National Engineering Laboratory for Cereal Fermentation Technology, Jiangnan University, Wuxi, China; ^3^Science Center for Future Foods, Jiangnan University, Wuxi, China; ^4^Jiangsu Provisional Research Center for Bioactive Product Processing Technology, Jiangnan University, Wuxi, China

**Keywords:** 2-keto-L-gulonic acid, *Gluconobacter oxydans*, promoters, L-sorbose, sorbose dehydrogenase

## Abstract

The acetic acid bacterium *Gluconobacter oxydans* is known for its unique incomplete oxidation and therefore widely applied in the industrial production of many compounds, e.g., 2-keto-L-gulonic acid (2-KLG), the direct precursor of vitamin C. However, few molecular tools are available for metabolically engineering *G. oxydans*, which greatly limit the strain development. Promoters are one of vital components to control and regulate gene expression at the transcriptional level for boosting production. In this study, the low activity of SDH was found to hamper the high yield of 2-KLG, and enhancing the expression of SDH was achieved by screening the suitable promoters based on RNA sequencing data. We obtained 97 promoters from *G. oxydans*’s genome, including two strong shuttle promoters and six strongest promoters. Among these promoters, P_3022_ and P_0943_ revealed strong activities in both *Escherichia coli* and *G. oxydans*, and the activity of the strongest promoter (P_2703_) was about threefold that of the other reported strong promoters of *G. oxydans*. These promoters were used to overexpress SDH in *G. oxydans* WSH-003. The titer of 2-KLG reached 3.7 g/L when SDH was under the control of strong promoters P_2057_ and P_2703_. This study obtained a series of gradient promoters, including two strong shuttle promoters, and expanded the toolbox of available promoters for the application in metabolic engineering of *G. oxydans* for high-value products.

## Introduction

*Gluconobacter oxydans* has been widely applied in the industrial production of L-sorbose from D-sorbitol (De Wulf et al., 2000), dihydroxyacetone from glycerol (De La Morena et al., 2020), 1-amino-L-sorbose from 1-amino-D-sorbitol (Schedel, 2000), and levan-type fructans from sucrose (Hövels et al., 2020). Furthermore, *G. oxydans* is also an excellent workhorse for the biosynthesis of 2-keto-D-gluconate (Li et al., 2016; Zhou et al., 2020), 5-keto-D-gluconate (Merfort et al., 2006), xylonic acid (Hahn et al., 2020; Shen et al., 2020), 5-keto-D-fructose (Battling et al., 2020; Hoffmann et al., 2020), and many other products (Deppenmeier et al., 2002; De Muynck et al., 2007). The wide applications of *G. oxydans* are mainly due to its unique dehydrogenases in the periplasm (Deppenmeier et al., 2002). Many protein engineering approaches have been used to improve the catalytic efficiency of these dehydrogenases, including enzyme immobilization (Kim et al., 2016), cofactor regeneration (Gao et al., 2019), ligand docking, and molecular dynamics simulations (Selvaraj et al., 2016). On the other hand, many metabolic engineering strategies of *G. oxydans* were based on the overexpression of related dehydrogenases or enzymes associated with the respiratory chains (Li et al., 2016; Yuan et al., 2016). However, the expression of key enzymes were often impeded by the accessible promoters.

Though the first genome of *G. oxydans* was reported in 2005 (Prust et al., 2005), only a few studies on the promoters of *G. oxydans* have been carried out. Generally, most studies directly selected some promoters from high expression level of genes. Nishikura-Imamura et al. (2014) cloned the putative promoter region of *G. oxydans* PQQ-dependent alcohol dehydrogenase (P_*adhAB*_) to overexpress 3-dehydroquinate dehydratase. Mientus et al. (2017) characterized promoters of six membrane-bound dehydrogenases of *G. oxydans* 621H, and used the constitutive promoter of the alcohol dehydrogenase and the glucose-repressed promoter of inositol dehydrogenase to construct a shuttle vector system. Though some progress were achieved in promoter discovery, it was hard to apply the promoters to other metabolic pathways because the promoters were relatively weak without systematic comparison. Saito et al., found some strong promoters of *G. oxydans*, such as P_*tufB*_, P_0169_, and P_264_ (Saito et al., 1997; Yuan et al., 2016; Blank and Schweiger, 2018). Moreover, Kallnik and Hu reported some promoters of different strengths in *G. oxydans* (Kallnik et al., 2010; Hu et al., 2015). Nevertheless, the available promoters are still insufficient, especially the strong promoters are highly needed to support engineering *G. oxydans* for their industrial application.

RNA sequencing (RNA-Seq) in the study of prokaryotic and eukaryotic organisms has become more accessible in the last decade (Poulsen and Vinther, 2018; Stark et al., 2019). It has become an excellent strategy to mine strong promoters in many microorganisms. Lee et al. (2015) screened a novel strong promoter P_*TN*0510_ from *Thermococcus onnurineus* by RNA-Seq and applied it to the production of H_2_. Liao et al. (2015) identified a strong promoter, P_*r*2_, from the RNA-Seq data of *Bacillus amyloliquefaciens* and verified it by measuring beta-galactosidase activity. Several studies about the transcriptome analysis of *G. oxydans* has been reported to reveal the secretion pathways of PQQ (Wan et al., 2017) and the response to osmotic and oxidative stress of 2-keto-L-gulonic acid (2-KLG) (Fang et al., 2020). Kranz et al. (2018) provided deep insights into the transcriptional landscapes of *G. oxydans* including promoters and other regulatory elements. However, no further experimental studies were performed to characterize these promoters and regulatory elements. Thus, RNA-Seq of *G. oxydans* WSH-003 was first conducted in this study, followed with the characterization of promoters by using mCherry as a report to compare the strength of the screened promoters.

2-KLG is an important precursor of vitamin C in industry (Wang et al., 2018). However, there are only a few *G. oxydans* strains that can produce 2-KLG naturally (Hoshino et al., 1990; Saito et al., 1998; Chen et al., 2019), although many sequenced *G. oxydans* possess the entire set of 2-KLG biosynthesis genes (Wang et al., 2018). In our previous study, we identified the key SDH from a *G. oxydans* that was able to naturally produce 2-KLG, and successfully constructed a high-throughput screening platform for an FAD-dependent SDH (Shan et al., 2020). Different from SSDHs from *Ketogulonigenium vulgare*, SDH from *G. oxydans* showed higher substrate specificity to L-sorbose and did not require PQQ as a cofactor (Saito et al., 1997; Wang et al., 2018). In the present study, a group of gradient promoters was identified and applied in the biosynthesis of 2-KLG in the strain *G. oxydans* WSH-003. The titer of 2-KLG reached 3.7 g/L when used the strongest promoter (P_2703_) to overexpress SDH. The results implied the low expression level of SDH may be the main problem for 2-KLG production in many *G. oxydans* strains. In conclusion, this study obtained a series of gradient promoters, and these promoters revealed promising prospects in metabolic engineering of *G. oxydans* for high-value products.

## Materials and Methods

### Genes, Plasmids, and Strains

*Escherichia coli* JM109 was used for plasmid construction. *G. oxydans* WSH-003 was used for PCR amplification of promoters and protein expression. *G. oxydans* ATCC 621H was used for PCR amplification of promoters. *G. oxydans* WSH-004 was screened in our previous research (Chen et al., 2019). *G. oxydans* WSH-003-Δ*gdh* was used for 2-KLG production. The plasmids p2-5 and pBBR1MCS-5 were used to overexpress mCherry (Li et al., 2020) and sorbose dehydrogenase in *G. oxydans*, respectively. All strains and plasmids are listed in Table 1. The nucleotide sequences of p2-5 and pBBR1MCS-5 were listed in Supplementary Table 5.

**TABLE 1:** Plasmids and strains used in this study.

Plasmids or strains	Characteristics	Sources
**Plasmids**		
p2-5	Km^R^, shuttle vector of *G. oxydans*, used for overexpressing of mCherry	Stored in Lab
pBBR1MCS-5	Gm^R^, shuttle vector of *G. oxydans*, used for overexpressing of sorbose dehydrogenase	Yuan et al., 2016
**Strains**		
*E. coli* JM109 *G. oxydans* WSH-003	Used for plasmid construction Cef^R^, used for PCR amplification of promoters and protein expression	Stored in Lab Hu et al., 2015
*G. oxydans* WSH-004	Cef^R^, used for PCR amplification of sorbose dehydrogenase	Chen et al., 2019
*G. oxydans* ATCC 621H	Cef^R^, used for PCR amplification of promoters	Prust et al., 2005
*G. oxydans* WSH-003-Δ*gdh*	Cef^R^ and Km^R^, deletion of glucose dehydrogenase (GenBank: AHKI01000025, from 1155 to 3575), used for 2-KLG producing	Stored in Lab

### RNA Sequencing and Data Analysis

The strain *G. oxydans* WSH-003 was cultured to mid-log phase in sorbitol medium (50 g/L sorbitol and 10 g/L yeast extract) at 30°C with shaking at 220 rpm. Then the cells were harvested and washed twice with PBS. The total RNA was extracted by RNeasy Mini Kit (Qiagen, Hilden, Germany), and ribosomal RNAs were removed by Ribo-Zero^TM^ rRNA Removal Kits (Epicentre, Wisconsin, United States). The RNA sequencing libraries were constructed by TruSeq RNA Sample Preparation Kit v2 (Illumina, California, United States) and sequenced on MiSeq (Illumina, California, United States) using MiSeq Reagent Kit v3 (Illumina, California, United States). RNA sequencing was performed by Shanghai Biotechnology Corporation (Shanghai Biotechnology Co., Shanghai, China). The abundance of transcripts was determined using bowtie2 (Langmead and Salzberg, 2012; Bolger et al., 2014) and cufflinks (Trapnell et al., 2010) by mapping the appropriate reads to the genome of *G. oxydans* WSH-003 (Gao et al., 2012).

### Genetic Operations

All the promoters were worked with a native ribosomal binding site (RBS), because it was hard to find a proper RBS with guaranteed strength. Promoters P_*tufB*_ and P_*dnak*_ and all the screened potential promoters were obtained by PCR amplification from the genomic DNA of *G. oxydans* WSH-003. Promoters P_264_ and P_*hp*0169_ were obtained by PCR amplification from the genomic DNA of *G. oxydans* ATCC 621H. The gene *sdh* was PCR-amplified from the genomic DNA of *G. oxydans* WSH-004. The gene *mCherry* was kept in our laboratory and obtained by PCR amplification. The *mCherry* gene was first ligated into the vector p2-5 to form the skeleton plasmid p2-5-mCherry by a one-step cloning kit (Takara, Dalian, China). Then different promoters were individually inserted into the plasmid p2-5-mCherry by the one-step cloning kit (Takara, Dalian, China). The *sdh* gene and gradient promoters were ligated into the vector pBBR1MCS-5 in the same way. All promoters and genes were verified by Sanger sequencing (Sangon Biotech, Shanghai, China). All vectors were constructed and amplified in the strain *E. coli* JM109. The vectors p2-5 and pBBR1MCS-5 were transferred by electroporation into *G. oxydans* WSH-003 (Zhang et al., 2010), which were selected using kanamycin and gentamycin, respectively. All primers are listed in Supplementary Table 1.

### Fluorescence Intensity Assay

Single colonies of *G. oxydans* WSH-003 were picked into 14 mL tubes containing 2 mL of sorbitol medium and cultured for 24 hours at 30°C with shaking at 220 rpm. Then 2% of these cultures were inoculated into a 250 mL flask containing 25 mL of sorbitol medium and cultured at 30°C with shaking at 220 rpm. The cell fluorescence and cell density (OD_600_) were measured every 4 hours on a Synergy H1 Hybrid Multi-Mode Microplate Reader (BioTek Instruments, Winooski, VT, United States) with excitation and emission wavelengths of 580 and 610 nm, respectively. The relative activity of mCherry was defined as the ratio of relative fluorescence unit (RFUs) divided by the optical density (OD_600_). The strain *G. oxydans* WSH-003 harboring p2-5-mCherry without promoters was used as the control.

### Culture Conditions for 2-KLG Production

The fermentation medium was formed with sorbitol medium (50 g/L sorbitol and 10 g/L yeast extract) containing 20 g/L CaCO_3_. Single colonies of *G. oxydans* WSH-003 were picked into 250 mL flasks containing 25 mL of sorbitol medium and cultured for 24 hours at 30°C with shaking at 220 rpm. Then, 10% of these cultures were inoculated into 250 mL flasks containing 25 mL of fermentation medium and cultured at 30°C with shaking at 220 rpm.

### Analysis of 2-KLG Production

The cell concentration was measured using a Microplate Reader (BioTek Instruments, Winooski, VT, United States). The concentrations of D-sorbitol, L-sorbose, and 2-KLG were detected by HPLC using an Aminex HPX-87H column (BioRad, Hercules, CA, United States) at 35°C with 5 mmol/L H_2_SO_4_ as the eluent at a flow rate of 0.5 mL/min (Chen et al., 2019).

### Statistical Analysis

The results were interpreted with mean values and its standard. The paired two-tailed Student *t* tests were performed to demonstrate statistically significant differences between data points. A p-value of ≤ 0.05 was thought to be statistically significant. For data illustration, bar charts with error bars were used.

## Results

### The RNA Sequencing Results

The isolated mRNAs of *G. oxydans* WSH-003 were subjected to high-throughput Illumina paired-end sequencing to obtain a global view of the transcriptome after removing ribosomal RNAs. The RNA sequencing data was submitted to sequence read archive (SRA) with the accession number of PRJNA706889. A total of 19.84 million mapped reads with an average length of 100 bp were obtained; 19.31 million reads were uniquely mapped to the genome of *G. oxydans* WSH-003, which represented a 500-fold coverage of the genome. The transcriptome data were analyzed with the software bowtie2 (Langmead and Salzberg, 2012; Bolger et al., 2014) and cufflinks (Trapnell et al., 2010). Only 188 genes were transcribed with transcript per million (TPM) values higher than 1000, and nearly 95% of the predicted 3545 genes were transcribed with TPM values below 1000 (Supplementary Table 2).

**FIGURE 1:**
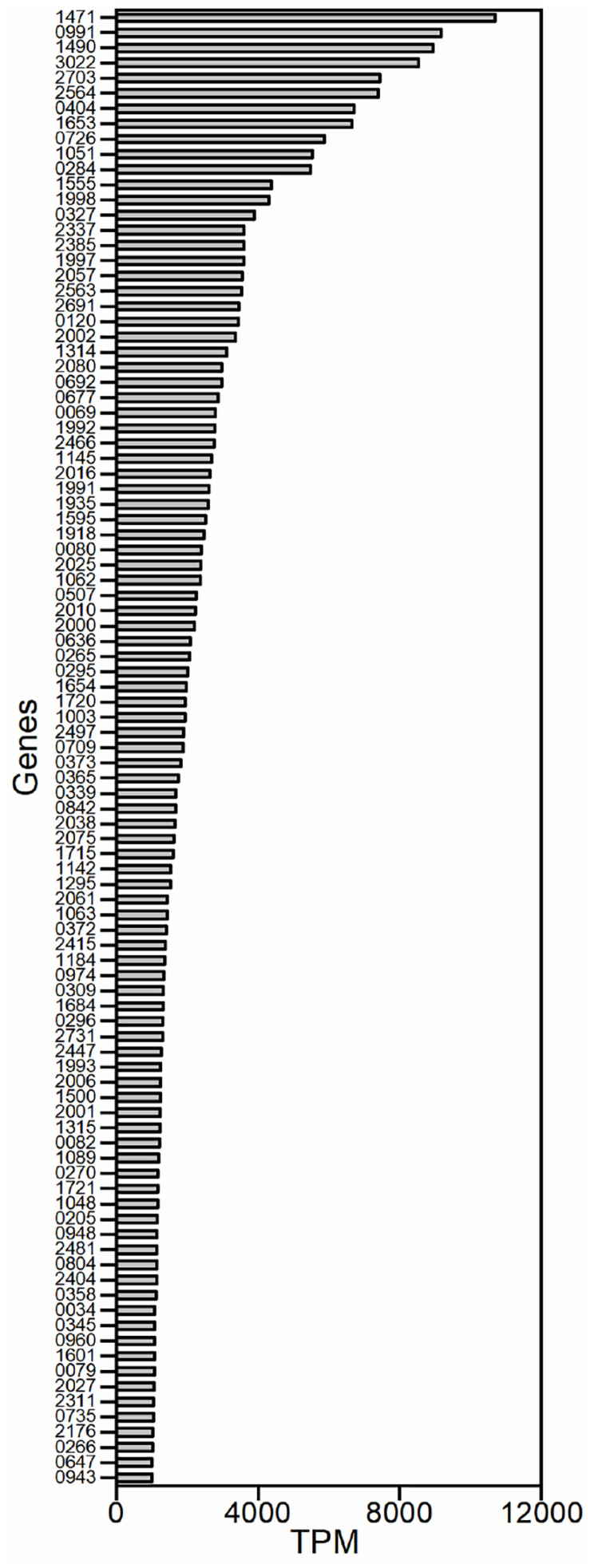
The strongly transcribed genes of *G. oxydans* WSH-003 obtained by RNA sequencing. The promoters were selected based on transcript per million (TPM) values of the transcriptome. The abscissa axis shows the TPM values. The ordinate axis shows the different genes in *G. oxydans* WSH-003.

Because we aimed to identify strong promoters in *G. oxydans*, the genes that exhibited strong transcriptional activity were studied. Genes were excluded from our analysis if they encoded tRNAs or lacked a RBS. Genes that belonged to a gene cluster were also excluded because the same promoter probably controlled their transcription. Gene clusters were defined as contiguous genes with similar functions and with spacers smaller than 50 nucleotides. A total of 97 potential promoters were obtained based on the TPM values in the transcriptome (Figure 1). The sequences around 500 bp upstream of the open reading frame (ORF) were chosen as the potential promoters because little information on the promoter elements of *G. oxydans* has been reported.

### Evaluation of Promoter Strength by Measuring mCherry Expression

**FIGURE 2:**
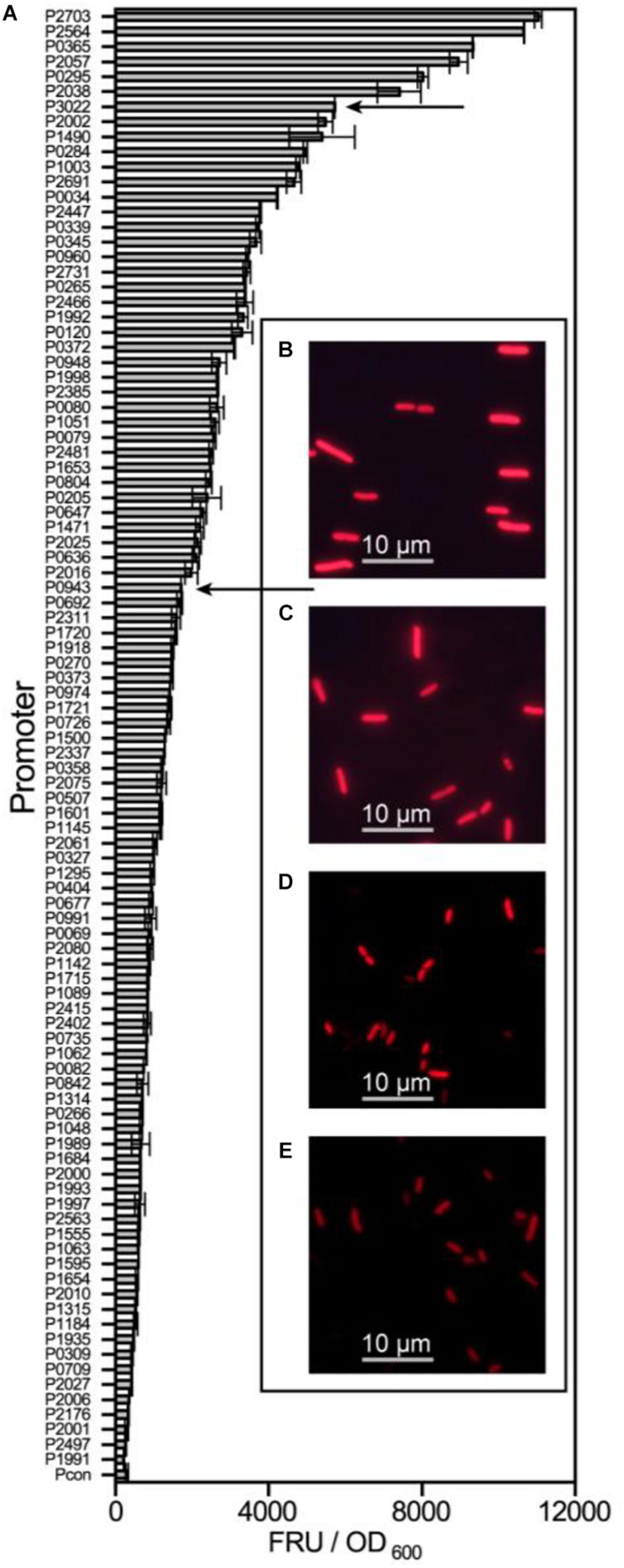
The strength determination of screened promoters of *G. oxydans* WSH-003. The strength of screened promoters and microscope pictures of two shuttle promoters. **(A)** The strength of screened promoters as measured by evaluating mCherry activity. The abscissa axis shows the relative activity of mCherry. The ordinate axis shows different promoters of *G. oxydans* WSH-003. Arrows indicate the two strong shuttle promoters P_3022_ and P_0943_. **(B)** *E. coli* JM109 expressing mCherry under the control of P_3022_. **(C)** *E. coli* JM109 expressing mCherry under the control of P_0943_. **(D)** *G. oxydans* WSH-003 expressing mCherry under the control of P_3022_. **(E)** *G. oxydans* WSH-003 expressing mCherry under the control of P_0943_.

All 97 potential promoters were transferred into *G. oxydans* WSH-003 to determine their strength by measuring mCherry expression. The fluorescence intensity was assayed every 4 hours. The highest value of relative mCherry activity was defined as the relative strength of the promoter. Most of the screened promoters showed remarkable intensity compared to the control (Figure 2). Among the promoters, the six strongest promoters were P_2703_, P_2564_, P_0365_, P_2057_, P_0295_, and P_2038_. Besides, it was found that most of the screened promoters had the highest strength at about 36 hours, when the strain was grown in stationary phase (Supplementary Figure 1).

**FIGURE 3:**
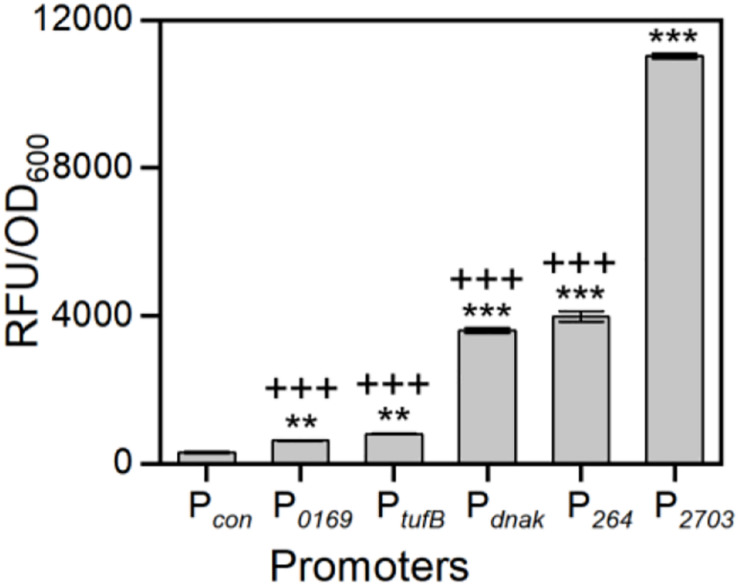
The comparison of screened strongest promoter with reported strong promoters. The abscissa axis shows different promoters of *G. oxydans*. P_*con*_ is the negative control. P_0169_ and P_264_ are reported strong promoters of *G. oxydans* ATCC 621H. P_*tufB*_ and P_*dnak*_ are reported strong promoters of *G. oxydans* WSH-003. P_2703_ is the strongest promoter screened in this study. The ordinate axis shows the relative activity of mCherry. **P < 0.01 compared with the P_*con*_ by two-tailed *t* test; ***P < 0.001 compared with the P_*con*_ by two-tailed *t* test. ^+++^*P* < 0.001 compared with the P_2703_ by two-tailed *t* test.

As mentioned previously, a few strong *G. oxydans* promoters have been reported (Hu et al., 2015; Li et al., 2016; Blank and Schweiger, 2018). To verify the strength of our strongest screened promoters, we also obtained four reported strong promoters (P_*tufB*_, P_*dnak*_, P_*hp*0169_, and P_264_) from the genomes of WSH-003 and ATCC 621H. Compared with these four reported strong promoters, the promoter P_2703_ has the highest strength, which was about 2.8-fold higher than that of P_264_ and about 3.1-fold higher than that of P_*dnak*_ (Figure 3). The results showed that P_2703_ was the strongest promoter discovered in *G. oxydans* at present. Interestingly, two strong shuttle promoters (P_3022_ and P_0943_) in *E. coli* and *G. oxydans* were discovered in this study. The *E. coli* JM109 containing the above plasmids showed visible red fluorescence (Supplementary Figure 2). When detecting the strength of these promoters in *G. oxydans* WSH-003, strong red fluorescence could also be observed (Figure 2). The shuttle promoters were often applied to the construction of shuttle vectors to express resistance genes or used to build a broad host expression system. To the best of our knowledge, such strong shuttle promoters have rarely been reported in *E. coli* and *G. oxydans*, although many shuttle promoters have been reported in other strains (Yang et al., 2018; Khan et al., 2020).

### Analysis of Screened Promoters

**FIGURE 4:**
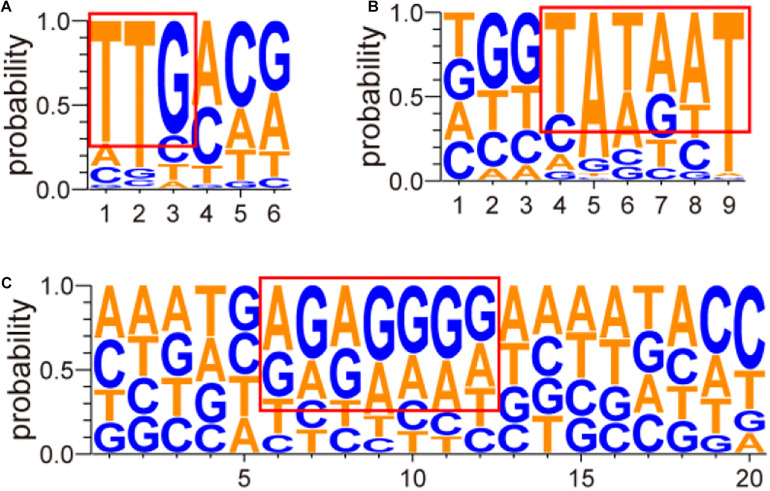
Sequence analysis of conserved nucleotides of screened promoters. The sequence analysis of conserved nucleotides of screened promoters was conducted using Softberry (Salamov and Solovyevand, 2011) and Neural Network Promoter Prediction (Reese, 2001). The abscissa axis shows the positions of nucleotides in different promoters. The 20 nucleotides before the initiation codon “ATG” or “GTG” were selected for the prediction of RBS. The ordinate axis shows the probability of each nucleotide. In the figure, **(A)** stands for the prediction result of the conserved region near position −35, **(B)** stands for the prediction result of the conserved region near position −10, and **(C)** stands for the prediction result of the conserved RBS.

The structures of these screened promoters were also analyzed in this study. The promoter sequences are listed in Supplementary Table 3. Analysis of the above promoter region and transcription start site was performed by Softberry^1^ (Salamov and Solovyevand, 2011) and Neural Network Promoter Prediction^2^ (Reese, 2001). The analyzed results are listed in Supplementary Table 4 and shown in Figure 4 as mapped by the website^3^ (Schneider and Stephens, 1990; Crooks et al., 2004). As shown in Figure 4, a “TTGnnn” region, with a highly conserved “TTG,” near position −35 and a “TATAAT” region near position −10 were found in the screened promoters. High frequencies of “A” or “G” were also observed at transcription initiation sites. At last, a region enriched in “A” and “G” was discovered at about eight nucleotides before the initiation codon “ATG” or “GTG” (Figure 4C), and many “AGGAg” regions were observed when strong promoters were analyzed (Supplementary Table 4).

### Application of Promoters for Improving the Production of 2-KLG

**FIGURE 5:**
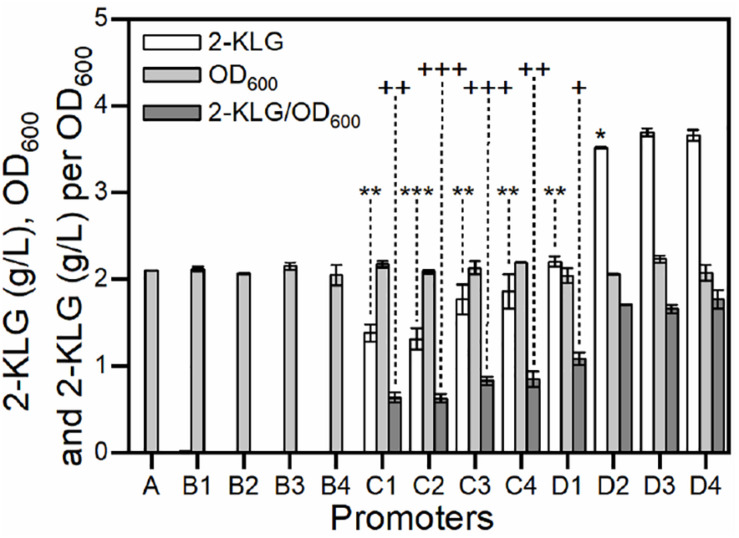
Effect of over-expressing SDH by gradient promoters on the growth of *G. oxydans* and the yield of 2-KLG. The abscissa axis shows different promoters controlling the expression of SDH in *G. oxydans*. **(A)** negative control; **(B1–B4)** weak promoters P_1142_, P_0991_, P_1295_, and P_0327_; **(C1–C4)** medium-strength promoters P_0647_, P_0205_, P_0804_, and P_1653_; **(D1–D4)** strong promoters P_3022_, P_2038_, P_2057_, and P_2703_. The ordinate axis shows the titer of 2-KLG (g/L), OD_600_ values and the titer of 2-KLG per OD_600_. *P < 0.05 2-KLG titer compared with D3 by two-tailed *t* test; **P < 0.01 2-KLG titer compared with D3 by two-tailed *t* test; ***P < 0.001 2-KLG titer compared with D3 by two-tailed *t* test; D3 and D4 are not statistically significant in 2-KLG titer with *p* = 0.0667. ^+^ *P* < 0.05 2-KLG titer per OD_600_ compared with D3 by two-tailed *t* test; ^++^*P* < 0.01 2-KLG titer per OD_600_ compared with D3 by two-tailed *t* test; ^+++^*P* < 0.001 2-KLG titer per OD_600_ compared with D3 by two-tailed *t* test; D2 and D3 are not statistically significant in 2-KLG titer per OD_600_ with *p* = 0.227; D4 and D3 are not statistically significant in 2-KLG titer per OD_600_ with *p* = 0.083.

Strain *G. oxydans* has potential in the biosynthesis of 2-KLG from D-sorbitol (Wang et al., 2018). It has been reported that SDH was an essential dehydrogenase in the conversion of L-sorbose to form 2-KLG in *G. oxydans* (Hoshino et al., 1990; Saito et al., 1998). In our previous study, it was also found that the rate-limiting step of the fermentation is the enzyme activity of SDH (Chen et al., 2019). In this study, a group of gradient promoters was selected to overexpress SDH in *G. oxydans* WSH-003. As shown in Figure 5, when weak promoters were used, the strains produced almost no 2-KLG; when medium-strength promoters were used, the strains could only synthesize 2-KLG with yields lower than 2.0 g/L; when strong promoters were applied, the strains could synthesize 2-KLG with yields of up to 3.7 g/L. The highest conversion yields achieved about 7% in mole number using strong promoters, almost twofold higher than that of those using medium-strength promoters. In addition, all the strains achieved similar biomass (Figure 5). Taken together, it can be concluded that the titer of 2-KLG increased with the enhancement of promoters or, in other words, 2-KLG production was positively related to the expression of SDH. These results demonstrated that the activity of SDH was indeed a rate-limiting step in the fermentation of 2-KLG.

## Discussion

*Gluconobacter oxydans* is an excellent host to produce 2-KLG, which is an essential precursor of vitamin C (Saito et al., 1998; Gao et al., 2014; Wang et al., 2018). With the help of gradient promoters screened, a series of 2-KLG-producing strains have been obtained. These 2-KLG-producing strains showed the highest titer when the strongest promoters were used, while almost no production when weak promoters were employed. It was consistent with the study of Saito et al. (1997) that the productivity of 2-KLG could be improved by optimizing promoters. The degradation of 2-KLG by a class of aldo-keto reductases was reported in some *G. oxydans* strains (Sugisawa et al., 1990; Saito et al., 1997) and *Aspergillus niger* (Kuivanen et al., 2017). That may be the reason that *G. oxydans* WSH-003 could not accumulate 2-KLG when the expression level of sorbose dehydrogenase was low. These results may explain why many *G. oxydans* strains possess the entire set of 2-KLG biosynthesis genes, but only a few strains produce 2-KLG naturally (Wang et al., 2018).

In this study, a group of promoters with different strengths was obtained based on RNA-Seq data of whole transcripts. The relative strength of these promoters covered a range of about 28 times, from 400 to 11,000, while reported promoters covered about 10 times, from 400 to 4000. Among them, the activity of the newly discovered strongest promoter P_2703_ was approximately threefold that of the reported strong promoters P_264_ and P_*dnak*_. Besides, two promoters P_0943_ and P_3022_ showed high activity in both *E. coli* and *G. oxydans*, revealing great potential in the construction of a shuttle expression system. The promoter region and the transcription start site of the screened promoters were also analyzed. The two strong shuttle promoters P_3022_ and P_0943_ had an excellent linear discriminant function (LDF) value, which may be the reason why these two promoters had high activities in both *E. coli* and *G. oxydans*.

In recent years, researchers have conducted many studies on the promoters of prokaryotes, especially model microorganisms such as *E. coli* (Schuller et al., 2020), *Bacillus subtilis* (Castillo-Hair et al., 2019), and *Corynebacterium glutamicum* (Dostalova et al., 2019). Based on these studies, researchers could have a detailed knowledge of these promoters’ structures and transcription factors. In this study, we found a “TTGnnn” region, with a highly conserved “TTG,” nearing position −35 and a “TATAAT” region nearing position −10, which was in accordance with the results in many other bacteria like *E. coli*. However, the “TATnnT” region nearing position −10 was not observed in the strong promoters of *G. oxydans* (Supplementary Table 4). This result is in agreement with the study of Kranz, and the main reason may be that the promoter motif is recognized by alternative sigma factors except σ^70^ (Kranz et al., 2018). The higher frequency of “A” or “G” at transcription initiation sites supported the theory that purine nucleotides are related to the increased transcription initiation rates (Mendoza-Vargas et al., 2009). Consistent with a prediction by Kranz et al. (2018) the conserved RBS motif “AGGAg” was also found in the strong promoters of *G. oxydans*.

With the development of synthetic biology, many methods can be applied to improve the strength of promoters, for example, randomization of the non-conserved region of the promoters (Siegl et al., 2013), error-prone PCR (Swagatika et al., 2019), hybrid or cascade promoters (Zhou et al., 2017), the design of RBS by RBS Calculator (Salis, 2011), and the use of a promoter library based on machine learning (Zhao et al., 2020). On the other hand, many other promoters like shuttle promoters and inducible promoters are also crucial in protein engineering and metabolic engineering. A strong shuttle promoter, P_*bs*_, for *B. subtilis*, *E. coli*, and *Saccharomyces cerevisiae* was constructed by Yang et al. (2018). Three broad-spectrum promoters (P_*bs*1_, P_*bs*2_, and P_*bs*3_) with different strengths, were generated by random mutation and characterized. In a recent study, a newly tunable L-arabinose-inducible P_*BAD*_ promoter was discovered to be useful in *G. oxydans* 621H, and the activity of this promoter was affected by the pH of the medium (Fricke et al., 2020). In summary, the identification of gradient promoters in this study expanded the toolbox of available promoters, and these promoters revealed promising prospects in metabolic engineering of *G. oxydans* for high-value products. With further research, more serviceable promoters of *G. oxydans* are expected to be discovered and constructed.

## Data Availability Statement

The data presented in the study are deposited in online repositories: https://www.ncbi.nlm.nih.gov/biosample/?term=SAMN18147321.

## Author Contributions

YC, LL, and SY performed the experiments and data analysis. YC and JZ wrote the manuscript and conceived the study. JL, JZ, and JC coordinated the project. All authors contributed to the article and approved the submitted version.

## Conflict of Interest

The authors declare that the research was conducted in the absence of any commercial or financial relationships that could be construed as a potential conflict of interest.

## Footnotes

1 http://www.softberry.com/

2 https://www.fruitfly.org/seq_tools/promoter.html

3 http://weblogo.threeplusone.com/
