# Supplementary material for: Identification of Gradient Promoters of Gluconobacter oxydans and Their Applications in the Biosynthesis of 2-Keto-L-Gulonic Acid
Source: Front Bioeng Biotechnol. 2021 Apr 9;9:673844. doi: 10.3389/fbioe.2021.673844 (PMC8064726; doi:10.3389/fbioe.2021.673844)

Supplemental materials Fig. S1 and Fig. S2.

Journal: Frontiers in Bioengineering and Biotechnology

Title: Identification of gradient promoters of *Gluconobacter oxydans* and their applications in the biosynthesis of 2-keto-_L_-gulonic acid

Author: Yue Chen^1,2,3,4^, Li Liu^1,2,4^, Shiqin Yu^1,2,3,4^, Jianghua Li^1,3^, Jingwen Zhou^1,2,4^*, Jian Chen^1,2^*

Author affiliation:

1 Key Laboratory of Industrial Biotechnology, Ministry of Education and School of Biotechnology, Jiangnan University, 1800 Lihu Road, Wuxi, Jiangsu 214122, China;

2 National Engineering Laboratory for Cereal Fermentation Technology, Jiangnan University, 1800 Lihu Road, Wuxi, Jiangsu 214122, China;

3 Science Center for Future Foods, Jiangnan University, Wuxi 214122, China;

4 Jiangsu Provisional Research Center for Bioactive Product Processing Technology, Jiangnan University, 1800 Lihu Road, Wuxi, Jiangsu 214122, China.

*Corresponding authors:

Jingwen Zhou, Jian Chen

Mailing address: School of Biotechnology, Jiangnan University, 1800 Lihu Road, Wuxi, Jiangsu 214122, China

Phone: +86-510-85914317, Fax: +86-510-85914317

E-mail: zhoujw1982@jiangnan.edu.cn, jchen@jiangnan.edu.cn.

Figures

Fig. S1. The strength of promoters at different times.

The strength of promoters was detected every four hours. The strength of promoters reached the highest activity at about 36 hours after inoculating and slightly dropped down at the next culturing.


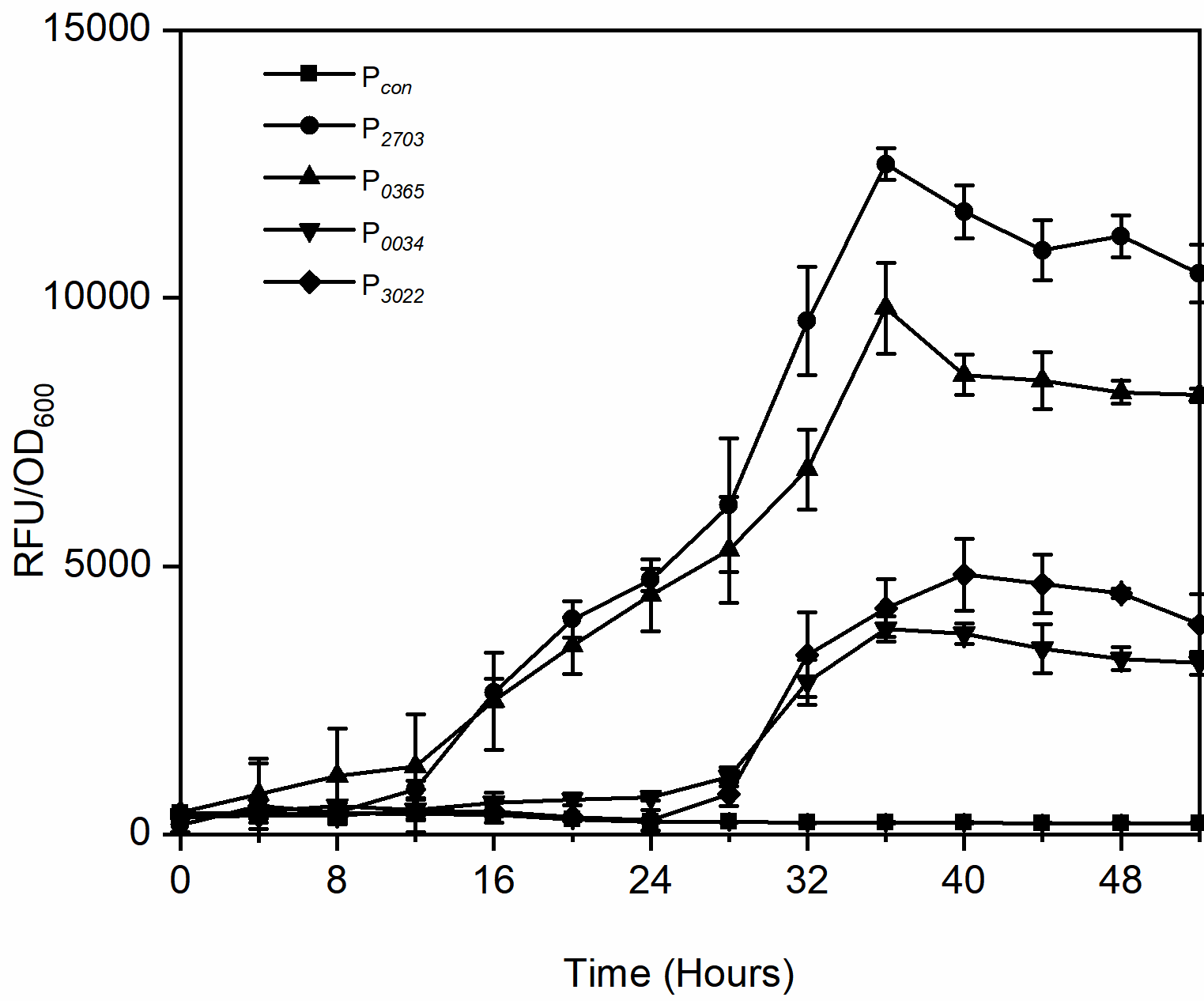


Fig. S2. The discovery of two shutter promoters P*_0943_* and P*_3022_*.

Two shutter promoters P*_0943_* and P*_3022_* were discovered when we extract plasmids from the *E. coli* JM109. The strains were centrifuged after culturing in LB medium for 12 hours at 37^o^C. The left figure is the strain expressing mCherry with P*_3022_*. The right figure is the strain expressing mCherry with P*_0943_*.


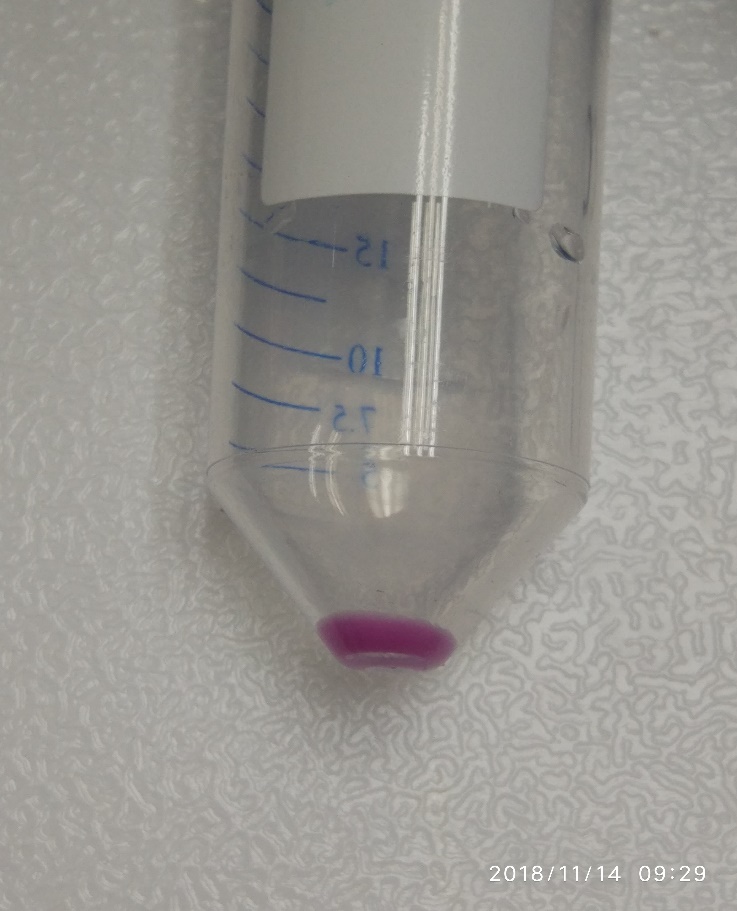

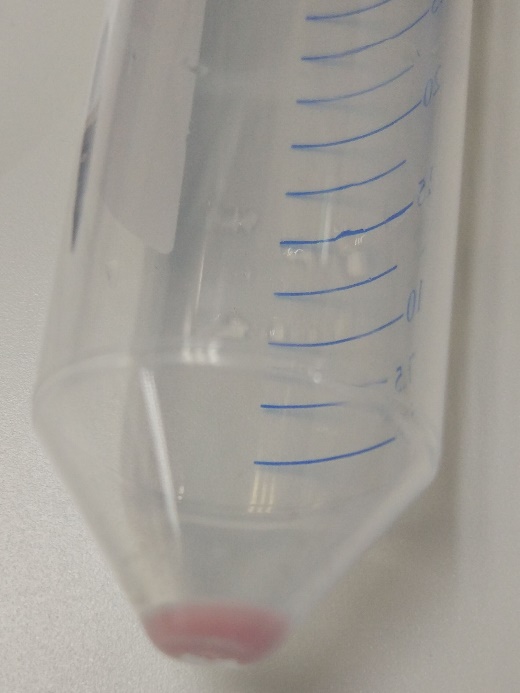

Supplement: Supplementary file 1 [file Data_Sheet_1.doc]
